# Role of topical beta blockers in regression of infantile capillary hemangioma

**DOI:** 10.12669/pjms.37.7.4317

**Published:** 2021

**Authors:** Murtaza Sameen Junejo, Rebecca, Maria Nazish Memon, Sajida Parveen Shaikh

**Affiliations:** 1 Dr. Murtaza Sameen Junejo, FCPS. Department of Ophthalmology, Liaquat University of Medical and Health Sciences, Jamshoro, Pakistan; 2 Dr. Rebecca, MBBS. Department of Ophthalmology, Liaquat University of Medical and Health Sciences, Jamshoro, Pakistan; 3 Dr. Maria Nazish Memon, FCPS, FCPS (Paediatirc Ophthalmology) Department of Ophthalmology, Liaquat University of Medical and Health Sciences, Jamshoro, Pakistan; 4 Dr. Sajida Parveen Shaikh, FCPS. Department of Ophthalmology, Liaquat University of Medical and Health Sciences, Jamshoro, Pakistan

**Keywords:** Capillary hemangioma, Superfical Hemangioma, Topical timolol maleate 0.5%

## Abstract

**Objective:**

To observe efficacy of Timolol maleate 0.5% by topical and surface application in infantile superficial capillary hemangioma of eyelid.

**Methods:**

This multi-centered clinical case series was carried out at Ophthalmology Department of Bilawal Medical College and Institute of Ophthalmology, LUMHS, Jamshoro from November 2019 to May 2020. We included 14 subjects. All the patients were subjected to detailed clinical examination. Before starting the topical beta blockers, the enrolled subjects had obtained the expert opinion by pediatrician to rule out any preexisting developmental cardio vascular disease. Topical beta blockers 0.5% drops were thus started with, against the ongoing finding of superficial capillary hemangioma of eyelid.

**Results:**

There was significant regression in size of infantile hemangioma after treating with topical timolol maleate 0.5%. We included 14 subjects in this study. Mean±SD age of patients was 4.94. Complete regression was seen in 08 subjects at the end of 12 weeks (64%) while 03 were completely cured at 08 weeks (21%) and 01 patient lost follow up with us. No significant ocular and systemic side effects were noted.

**Conclusion:**

Topical timolol maleate 0.5% can be the first-line treatment modality for superficial capillary hemangiomas due to its better safety and efficacy.

## INTRODUCTION

Infantile hemangiomas (IH) are benign proliferation of endothelial cells mostly arising in first 08 weeks of life.^1^,^2^ IH are most frequent benign tumors of infancy. The incidence in one year old children is 5-10% in first year of life. Preterm infants with low birth weight have relatively higher risk of about 23%.^2^

The cycle of disease is characterized by early proliferative phase, followed by involution and results in complete regression in most cases. During first few months aged, the lesion grows aggressively, followed by stabilization. Involution is complete in most youngsters by four years of age.^3^ Mostly lesions are benign, regress spontaneously, but approximately 10% often site dependent can cause serious complications like Astigmatism and Amblyopia. Thanks to cosmetic disfigurement, parental distress, and morbidity, appropriate management are much needed.^4^,^5^

Previously hemangiomas were treated with ultrapotent topical corticosteroids, which were potent but were causing side effects like skin hypopigmentation and atrophy.^6^-^8^ Imiquimod 5% cream topically has been used as an alternate but it also has side effects like crusting and scars.^9^-^12^

Topical β-blockers timolol maleate 0.5% are best substitute in managing superficial IHs asthey will improve the therapeutic efficacy and reduce systemic adverse effects of beta blockers.^13^However, systemic propranolol with a dose of two mg/kg/day is additionally used but can cause adverse systemic side effects.^14^,^15^

Topical timolol has been reported to inhibit the expansion and promote regression of superficial IH’s.^16^ So during this study we shall evaluate the safety and efficacy of 0.5% timolol maleate solution for superficial IH in early age bracket.

## METHODS

This multi-centered case series was conducted at Bilawal Medical College and Institute of Ophthalmology, LUMHS, Jamshoro from November 2019 to May 2020 after approval from ethical committee (LUMHS/REC/-828). Fourteen patients aged two months to 18 months were included during this study after an informed written consent.

All the subjects underwent detailed ocular and systemic examination and patients having superficial capillary hemangioma of eyelid were included. Subjects having hypertension, visceral involvement, respiratory problem & cardiovascular abnormalities were excluded from the study. Opinion of pediatrician to rule out developmental cardiovascular abnormalities was obtained before the commencement of treatment. Topical Timolol Maleate 0.5% 2 drops were applied over the lesion twice each day. Follow up was done at two weeks, eight weeks and 12 weeks respectively during which color and size of lesion were assessed. Statistical analysis was through with help of SPSS version 21.0. p-value was not calculated due to single group study.

## RESULTS

Fourteen (14) subjects received timolol maleate with a total mean±SD age of 4.94 (Male = 6.5 and female mean age = 4.08 months, respectively. Gender distribution of patients was 64% male and 36% female. The most common indication of treating IH’s was to prevent cosmetic disfigurement, ulceration, astigmatism and amblyopia. Clinical characteristics of patients are mentioned in Table-1. Complete regression of lesion was observed in 08 subjects at 12 weeks (64%), while 03 (21%) children got regression at 08 weeks. Unfortunately, one child developed ulceration at 02 weeks of start of timolol maleate (7%) whereas one got regression at 02 weeks and one patient lost follow up with us (7%) mentioned in Chart-II. No adverse ocular and systemic effects were seen. Photographs of patients taken after consent, before and after treatment are given in Fig.1 and 2.

**Table I:** Clinical characteristics of patients with capillary hemangioma.

*Characteristics*	*Value*
Age (Months)	4.94 months
***Gender***	
Male	5 (35.7 %)
Female	9 (64.3%)
***Regression***	
02 weeks	1 (7%)
08 weeks	3(21%)
12 weeks	8(64%)

**Chart.1:**
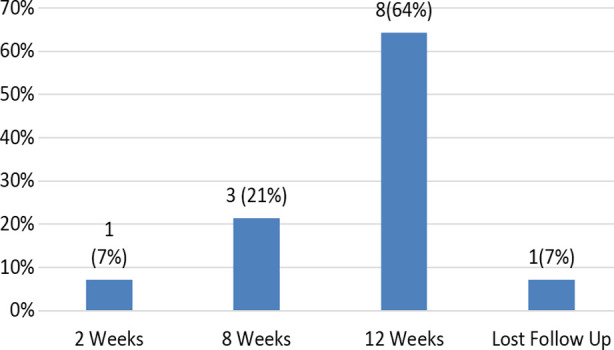
Regression of hemangioma after treatment with Timolol Maleate(n=14).

## DISCUSSION

Inafantile hemangiomas grow in early period of life that mostly resolves spontaneously. The proliferative phase commences during a week or two of life, that’s followed by a plateau phase. The involution phase is noticed to be commenced at a primary year of life and remains until 4-6 years aged. In our study, the initial age for the looks of IH’s was eight weeks, as compared with previous studies. Xu et al found that the therapeutic response of youngsters who started treatment with propranolol ointment had a big difference.^10^ Yu et al. noted that patients treated with timolol before the age of 06 months had higher regression rates. This is often in lieu with our study results.

**Case-1:**
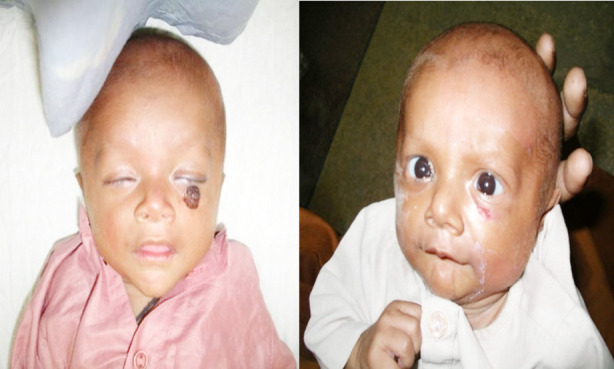
Before treatment 08 weeks after treatment

**Case-2:**
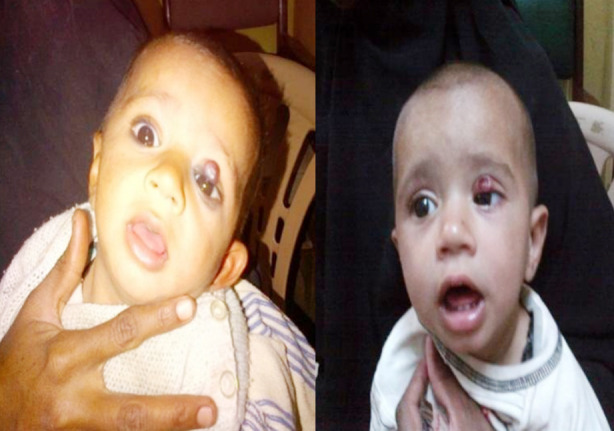
Before Treatment 02 weeks after treatment (LEFT EYE) (Ulcerat ed)

**Case-3:**
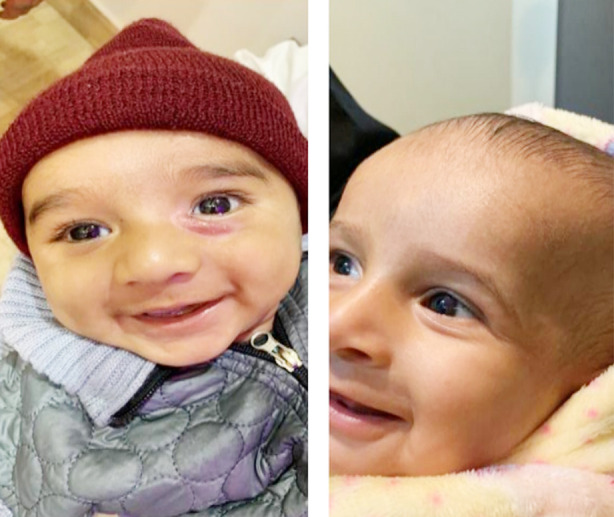
Before Treatment 08 weeks after (Left Eye) tre atment

Ariwibowo, Danarti et al. and associates found that Timolol maleate 0.5% the answer was better than corticosteroids in reducing the dimensions of superficial IH.^10^,^14^,^17^-^24^ Our study also showed reduction in size,colour and shape of superficial infantile hemangiomas in approximately 08-12 weeks after the initiation of treatment.

Regression of IH’s using 0.5% timolol maleate drops occurred before spontaneous regression which occurred at 9-12 months.^18^,^25^ In our study, there was complete regression in 08 cases upto 12 weeks while 03 cases regressed at 08 weeks from the onset of treatment. Spontaneous regression of IH’s in comparison with hemangiomas treated with timolol maleate 0.5%, morphological differences were observed. In later the regression of endothelial cells with apoptosis was observed.^24^,^25^ We didn’t observe the histological changes in our studies but the morphological outcomes were noteworthy and appreciable.

Despite our success of treating IH’s with topical timolol maleate 0.5%, the underlying mechanism of timolol maleate remains elusive. Further work should be administered and comparison of regression between children and adults should be investigated on large scale.

### Limitations of the study

we could not consider the presence of multiple and deep hemangiomas and their regression with topical timolol maleate 0.5 %. Moreover, we measured short term post treatment effect of 12 weeks.

## CONCLUSION

Topical timolol maleate 0.5% instead of oral propranolol could be the first-line approach for superficial capillary hemangiomas due to its good efficacy and improved safety. However, a comparative study between children and adult’s regression of hemangiomas must be conducted and evaluated at a larger scale.

### Authors Contribution:

**JMS:** conceived, designed and did statistical analysis & editing of manuscript, is responsible for integrity of research.

**JMS, R, MMN:** did data collection and manuscript writing.

**SPS:** did review and final approval of manuscript.
